# Early phase resolution of mucosal eosinophilic inflammation in allergic rhinitis

**DOI:** 10.1186/1465-9921-11-54

**Published:** 2010-05-09

**Authors:** Lena Uller, Cecilia Ahlström Emanuelsson, Morgan Andersson, Jonas S Erjefält, Lennart Greiff, Carl G Persson

**Affiliations:** 1 Department of Experimental Medical Science, Lund University, Lund, Sweden; 2 Department of Otorhinolaryngology, Lund University Hospital, Lund, Sweden; 3 Department of Clinical Pharmacology, Lund University Hospital, Lund, Sweden

## Abstract

**Background:**

It is widely assumed that apoptosis of eosinophils is a central component of resolution of allergic airway disease. However, this has not been demonstrated in human allergic airways in vivo. Based on animal in vivo observations we hypothesised that steroid-induced resolution of human airway eosinophilic inflammation involves inhibition of CCL5 (RANTES), a CC-chemokine regulating eosinophil and lymphocyte traffic, and elimination of eosinophils without evident occurrence of apoptotic eosinophils in the diseased tissue.

**Objective:**

To determine mucosal eosinophilia, apoptotic eosinophils, general cell apoptosis and cell proliferation, and expression of CCL5 and CCL11 (eotaxin) in human allergic airway tissues *in vivo *at resolution of established symptomatic eosinophilic inflammation.

**Methods:**

Twenty-one patients with intermittent (birch and/or grass) allergic rhinitis received daily nasal allergen challenges for two seven days' periods separated by more than two weeks washout. Five days into these "artificial pollen seasons", nasal treatment with budesonide was instituted and continued for six days in a double blinded, randomized, placebo-controlled, and crossover design. This report is a parallel group comparison of nasal biopsy histochemistry data obtained on the final day of the second treatment period.

**Results:**

Treatments were instituted when clinical rhinitis symptoms had been established. Compared to placebo, budesonide reduced tissue eosinophilia, and subepithelial more than epithelial eosinophilia. Steroid treatment also attenuated tissue expression of CCL5, but CCL11 was not reduced. General tissue cell apoptosis and epithelial cell proliferation were reduced by budesonide. However, apoptotic eosinophils were not detected in any biopsies, irrespective of treatment.

**Conclusions:**

Inhibition of CCL5-dependent recruitment of cells to diseased airway tissue, and reduced cell proliferation, reduced general cell apoptosis, but not increased eosinophil apoptosis, are involved in early phase steroid-induced resolution of human allergic rhinitis.

## Background

Airway tissue signs of established inflammation in asthma and allergic rhinitis include increased mucosal tissue cell turnover, eosinophilia, and increased chemokine production. Institution of steroid treatment eventually reduces both symptoms and eosinophilic inflammation in allergic airways diseases [1]. However, early resolution effects *in vivo *of this mainstay class of airway drugs are incompletely understood.

In recent years, based largely on observations *in vitro*, the view that established airway tissue eosinophilia is resolved through steroid-induced apoptosis of these cells has been widely accepted [2-5]. Unexpectedly, therefore, we and others observed that steroid-treatment resolved established airway-pulmonary eosinophilic inflammation in murine *in vivo *models without inducing any detectable apoptosis of tissue eosinophils [6-8]. At neither spontaneous nor steroid-induced resolution were apoptotic eosinophils seen in the tissues *in vivo *[7,8]. Although these data were at variance with predictions made from *in vitro *experiments they were compatible with publicised human and animal *in vivo*-information in the field of interest [9]. New data also emerged demonstrating that airway tissue eosinophils could efficiently and non-injuriously be eliminated by extra-apoptotic routes [7,9,10]. It is of note that the previous studies had not examined human diseased airways during steroid-induced resolution of allergic inflammation. This aspect is essential because at the early phase of such resolving tissue eosinophilia the chance of detecting apoptotic eosinophils would be the greatest.

Studies involving allergic animals and humans demonstrate broad anti-inflammatory airway effects when steroids are given as prophylactic treatment prior to allergen exposure [8,11]. By contrast, observations in animals indicate that steroids given to airways with already established allergic inflammation and remodelling may not exhibit the same wide range of early effects [8,12]. Prophylactic administration of a steroid thus inhibited allergen challenge-induced up-regulation of CCL11 (eotaxin) and CCL5 (RANTES) along with attenuation of several other CC-chemokines in mouse lungs [8]. However, using the same animal model and instituting the same steroid treatment after the allergic eosinophilic inflammation had been established, merely one lung tissue chemokine, CCL5, was significantly inhibited [8]. This latter effect was associated with steroid-induced resolution of the eosinophilic inflammation. Similar to several other eosinophil chemo-attractants, CCL5, a key regulator of airway eosinophils and lymphocytes, has been demonstrated in biopsies and lavage fluids obtained from human airways in allergic disease [13-17]. Supporting the possibility that an anti-CCL5 action is involved in therapeutic effects of steroids, Castro *et al*. further observed that CCL5 became significantly increased in asthmatic airway tissues along with worsening of the disease evoked by stopping steroid treatment [17]. However, it is not known whether early steroid-induced resolution of airway allergic inflammation involves any specific reduction of CCL5 in the diseased human tissues.

The human nose provides opportunities for the study of inflammatory processes germane to the respiratory tract mucosa [18]. Using a validated repeat allergen challenge-model closely mimicking seasonal allergic rhinitis [19,20], we have here studied early phase resolution of airway eosinophilic inflammation with and without steroid treatment. The features of this model include establishment of consistent around-the-clock disease symptoms along with eosinophilic inflammatory processes. In the present study, we report on tissue indices of inflammation assessed in biopsies obtained at one point in time (parallel group analysis) at the end of a crossover exploratory study of early resolution of symptomatic rhinitis. We have focused on epithelial and subepithelial eosinophilia, expression of CCL11 and CCL5, and occurrence of apoptotic and non-apoptotic eosinophils in the human nasal mucosa with resolving tissue eosinophilia. In addition, inflammation as reflected by mucosal cell turnover indices has been examined.

## Methods

### Study design and ethical approval

**Figure 1:**
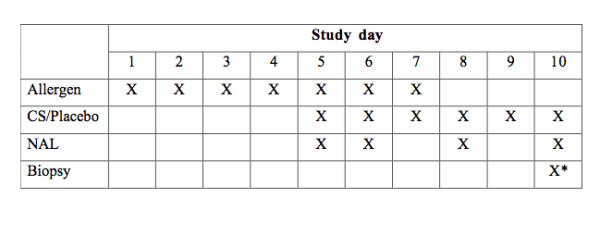
**Scheme of allergen challenges, budesonide (corticosteroid, CS) treatment, and sampling representing the challenge/treatment series**. The biopsies*, which are the focus of the present analysis, were obtained only on the final study day (Study day 10 of the second series of allergen challenges).

Patients with seasonal (intermittent) allergic rhinitis were subjected to daily nasal allergen challenges for two seven days' periods outside the pollen season. Five days into the "artificial pollen season" [19,20] nasal treatment with budesonide was instituted in a double blinded, randomized, placebo-controlled, and crossover design and continued for six days. (Fig.1). Nasal symptoms were monitored. On Study days 5, 6, 8, and 10 of each allergen challenge series, nasal lavage was carried out. However, the focus of the present report was on the analysis of nasal biopsies obtained on the final day of the second treatment period (Study day 10). This latter parallel-group comparison involved ten patients on treatment with budesonide and eleven patients on treatment with placebo. The human study was in accordance with the Helsinki Declaration, and ethical approval was obtained by the Ethical Committee at Lund University Hospital (reference number: LU504-01). All patients gave written consent prior to participate in the study.

### Patients

Twenty-one patients were recruited to the study (15 males and 6 females). The median age was 24 (range 20-41). Inclusion criteria were a history of strictly intermittent (birch and/or grass) allergic rhinitis for at least two years, a positive skin-prick test to birch and/or grass pollen allergen, and a need for medical treatment at seasonal allergen exposure. Exclusion criteria were asthma, a history of perennial (persistent) allergic rhinitis or a positive skin prick test to perennial allergens, and other chronic nasal disease or major nasal structural abnormalities. The only permitted drug treatment was rescue medication provided by the investigator (loratadine, 10 mg tablets, Schering-Plough).

### Allergen-challenge

In order to identify an individual, tolerable, yet symptom-producing dose for the nasal allergen-challenge series, a titration procedure was performed. Increasing doses of birch or timothy pollen allergen were administrated at 10-min intervals using a spray-device delivering 100 μl per actuation (Aquagen, ALK, Horsholm, Denmark). One puff was sprayed into each nostril resulting in effective doses of 100, 300, 1000, and 3000 Standard Quantity-Units (SQ-U) per cavity. This scheme was followed until a response of 5 sneezes or a score of 2 or more, on a scale from 0 to 3, for either of the symptoms blockage or rhinorrhea. The dose that produced this effect was chosen for the daily challenges.

### Study drug

The subjects were treated with budesonide (AstraZeneca R&D, Lund, Sweden) and placebo. An aqueous suspension of budesonide (1.28 mg/ml) was provided in glass bottles fitted with mechanical spray-pump delivering 64 μg budesonide per actuation. Placebo was provided in identical glass bottles and with identical spray pumps. The subjects were given two actuations into each nostril of either budesonide (total daily dose: 256 μg) or placebo once daily for six days, starting on Study day 5 of the allergen challenge series. The first dose of study drug/placebo was taken at the clinic under supervision of the investigator and the subsequent doses were taken at home every morning.

### Symptoms and nasal lavage

During the study period, nasal symptoms were scored in the morning (before intake of the drug), post challenge, and in the evening. The score in the morning and evening reflected the last 12 hours and was entered into a diary. The symptoms blocked nose, runny nose, and sneezing/itching were each scored on a 4-graded scale: 0 = no, 1 = mild, 2 = moderate and 3 = severe symptoms. Symptom scores for morning and evening recordings, respectively, were added to a total nasal symptom score (TNSS, range 0-9). Nasal saline lavages were carried out on Study days 5, 6, 8, and 10 using a nasal pool-device [21]. On Study days 5 and 6, these lavages were carried out before the daily allergen challenge. In the present study, the volume of the pool-fluid was 15 ml and the dwell time 5 min. The samples were centrifuged (325g, 10 min, 4°C) and samples were obtained from the supernatant and frozen (-30°C). CCL5 and CCL11 were measured with ELISAs (R&D Systems, Minneapolis, MN).

### Nasal biopsy

Two nasal biopsies were obtained from each patient at the end of the second treatment period (Study day 10). Topical anaesthesia and mucosal decongestion was achieved by administration of tetracain (20 mg/ml) and adrenalin (0.1 mg/ml) first by a spray-device and then by a cotton swab. In addition, ten min later, a mixture of carbocain (10 mg/ml) and adrenalin (5 mg/ml) was injected into the inferior nasal turbinate. Using a cutting forceps with a 3 mm drilled out punch, a biopsy was taken from the inferior turbinate about 5 mm from its anterior margin. The biopsies were directly frozen in TissueTek (Sakura Finetek Europe, Zoeterwoude, The Netherlands) and stored at -80ºC.

### Histological analyses of nasal biopsies

#### Histochemical detection of eosinophils

Eosinophils were detected by histochemical visualisation of cyanide-resistant eosinophil peroxidase as previously described [7,22]. Eosinophils were identified by their dark brown reaction product and quantified in the epithelial lining and the lamina propria as numbers of eosinophils/0.1 mm^2 ^tissue area and eosinophils/mm^2^, respectively. The whole biopsy was subjected to analysis in a blinded manner.

#### Detection of apoptotic cells with the TUNEL-technique

Apoptosis was visualized using the *in situ *TUNEL-technique that we previously have validated in animal and human airway tissues [7,22]. Cryosections (5-10 μm) were directly fixated in 2% paraformaldehyde and then pretreated with proteinase K (20 μg/ml) (Sigma, Stockholm, Sweden) for 15 min at room temperature. DNA strand breaks obtained during apoptosis were detected by enzymatic labelling of free DNA termini using an *in situ *apoptosis kit (ApopTag fluorescein, Intergen, NY). Thymus from corticosteroid-treated rats was used as a positive control. No staining was evident in negative controls, i.e., when the Tdt-enzyme was omitted. Slides were counterstained with propidium iodide to reveal pyknotic nuclei as well as the total number of cells. Apoptotic cell nuclei were stained intensely green and quantified as total apoptotic cells/mm^2^. Apoptotic eosinophils were defined as both chromotrope 2R-positive and TUNEL-positive cells exhibiting apoptotic morphology, i.e., small cells with condensed nuclei.

#### Immunostaining for proliferating cells using light microscopy

Cryosections were incubated over night with a monoclonal mouse anti human Ki-67 antibody (dilution 1:80, clone Ki-S5, Dako, Glostrup, Denmark) and visualised by a secondary goat anti-mouse FITC antibody (1:200, Jackson Immuno Research, West Grove, PA). Proliferating epithelial cells were quantified as number of cells/0.1 mm basement membrane.

#### Immunostaining for CCL5 and CCL11

Cryosections (5-10 μm) were directly fixated in ice-cold 2% formaldehyde and washed in EBSS buffer-saponin. A biotinylated anti-human CCL5 antibody (2.5 μg/ml, BAF 278, R&D Systems, Abingdon, UK.) and a monoclonal anti-human CCL11 antibody (5 μg/ml, MAB320, R&D Systems, Abingdon, Systems, UK.) were used and stained according to manufactures instructions. Haematoxylin was used as background staining. Sections were examined with a standard light microscope (Bx-60, Olympus, Tokyo, Japan) in a blinded manner and photomicrographs were taken with a 10x objective and saved in TIFF-format for analysis of Integrated Optical Density (IOD) using the Image-Pro Plus 4.5 software (Media Cybernetics, Silverspring, MD, USA) as previously described [23]. Image-pro Plus software was used to calculate stained area (μm^2^) and Integrated Optical Density (IOD) (area x average density) for CCL5 and CCL11.

### Statistics

Symptom scores, lavage fluid indices, and tissue indices were considered non-parametrically distributed. Differences between groups receiving treatment with budesonide and placebo were examined using the Mann Whitney U-test. P-values <0.05 was considered statistically significant. Paired analyses within each treatment group were carried out using the Wilcoxon Signed Rank test. Histology data is presented as mean ± SEM. Symptoms and lavage data in Table 1 are presented as median and interquartile range.

**Table 1:** Nasal symptom scores and cytokine levels in nasal lavage fluid.

	Treatment		
	
	Placebo (n = 11)	Budesonide (n = 10)	p-value
***Symptoms***			
- Morning TNSS	1.0 (1.0)	1.0 (2.0)	0.48
			
***Nasal lavage***			
- CCL5 (pg/ml)	8.5 (9.0)	10.0 (27.6)	0.14
- CCL11 (pg/ml)	8.8 (4.5)	11.0 (13.1)	0.17

Median (IQR) total nasal symptom scores, and lavage fluid levels of CCL5 (RANTES) and CCL11 (eotaxin) on Study day 10 of the second treatment series, i.e., on the day the biopsies were obtained. Data were analysed using the Mann Whitney test (unpaired comparisons). No statistically significant differences were observed between the treatments.

## Results

### Allergen challenges

Thirteen patients received nasal challenges with timothy-pollen allergen and 8 with birch-pollen allergen. The individual dose-titration resulted in that 4, 3, and 6 subjects received daily challenges with 100, 300, and 1000 SQ-U of timothy allergen, respectively and 4, 2, 1, and 1 subjects received 100, 300, 1000 and, 3000 SQ-U of birch allergen. Rescue medication (loratadine) was used by patients in both the placebo group (1 patient taking 4 tablets, 3 patients taking 1 tablet each) and budesonide group (2 patients taking 1 tablet each) during days 5- 8. Thus, no antihistamine medication was taken during the last 48h prior to sampling the biopsies.

### Symptoms and lavage data

The challenges produced mild rhinitis symptoms: For example, evening total nasal symptoms on Study day 4 (placebo and budesonide groups together prior to any treatment had begun) were scored 1.7 compared with evening symptoms on Study day 1 scored of 1.3. Budesonide reduced symptoms compared with placebo during Study days 5-10, but these changes failed to reach statistical significance (paired analysis involving both treatment periods, data not shown). At the time when the biopsies were obtained, symptoms also did not differ between the treatment groups (Table 1, parallel group analysis). During the present early phase of resolution lavage fluid levels of CCL5 and CCL11 did not differ between budesonide and placebo (paired analysis involving both treatment periods, data not shown, and parallel group analysis Study day 10, Table 1).

### Airway tissue eosinophilia

**Figure 2:**
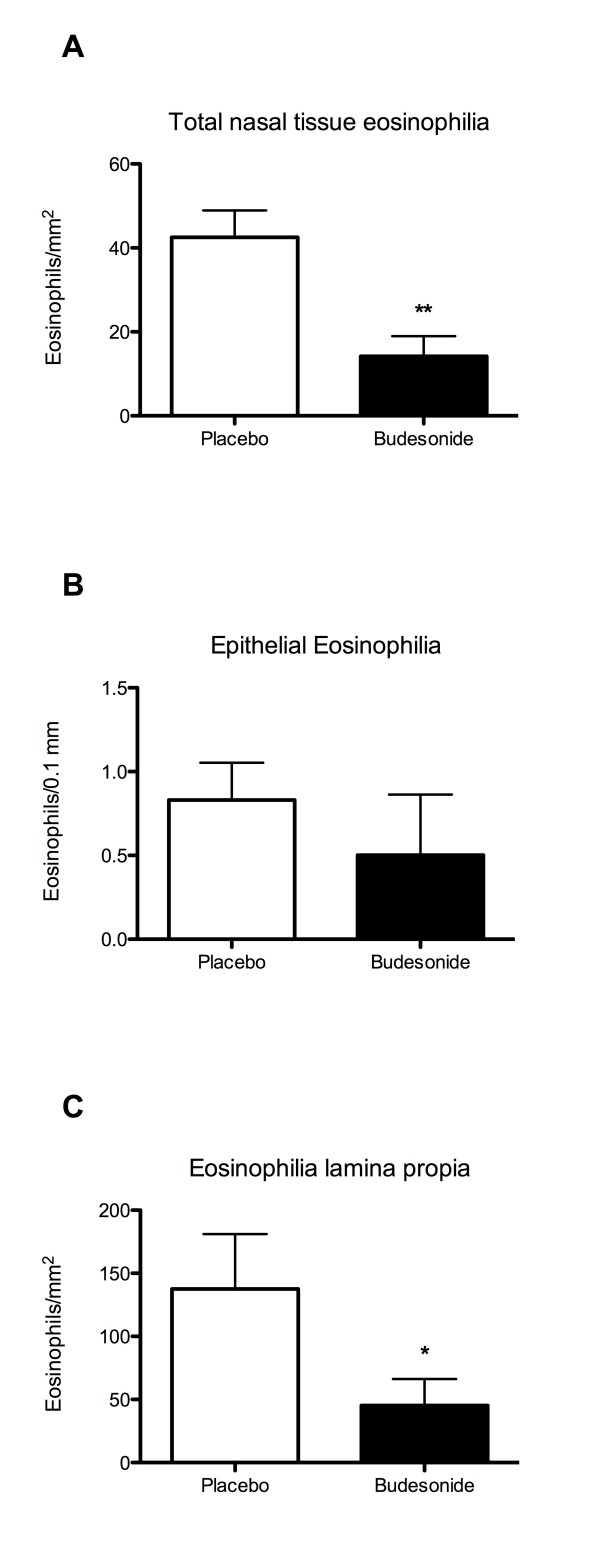
**Airway tissue eosinophilia in nasal biopsies during resolution phase **The total number of eosinophils in nasal biopsies was reduced after budesonide treatment (A). Eosinophils localised in the epithelial lining was not significantly reduced by budesonide treatment (B). The reduction was more pronounced in the subepithelial tissue (lamina propria; C). Data presented is mean ± SEM. (* Denotes p < 0.05 and ** denotes p < 0.01).

In biopsies obtained from placebo-treated subjects, a marked epithelial- and sub-epithelial eosinophilia remained three days after cessation of the allergen-challenges (Fig. 2). In budesonide-treated individuals, the total nasal tissue eosinophilia was reduced compared to placebo treatment (Fig. 2A). The epithelial eosinophilia was not significantly reduced by budesonide treatment (Fig. 2B) but the eosinophilia beneath the epithelium was significantly reduced (Fig. 2C).

### Apoptosis and proliferation

**Figure 3:**
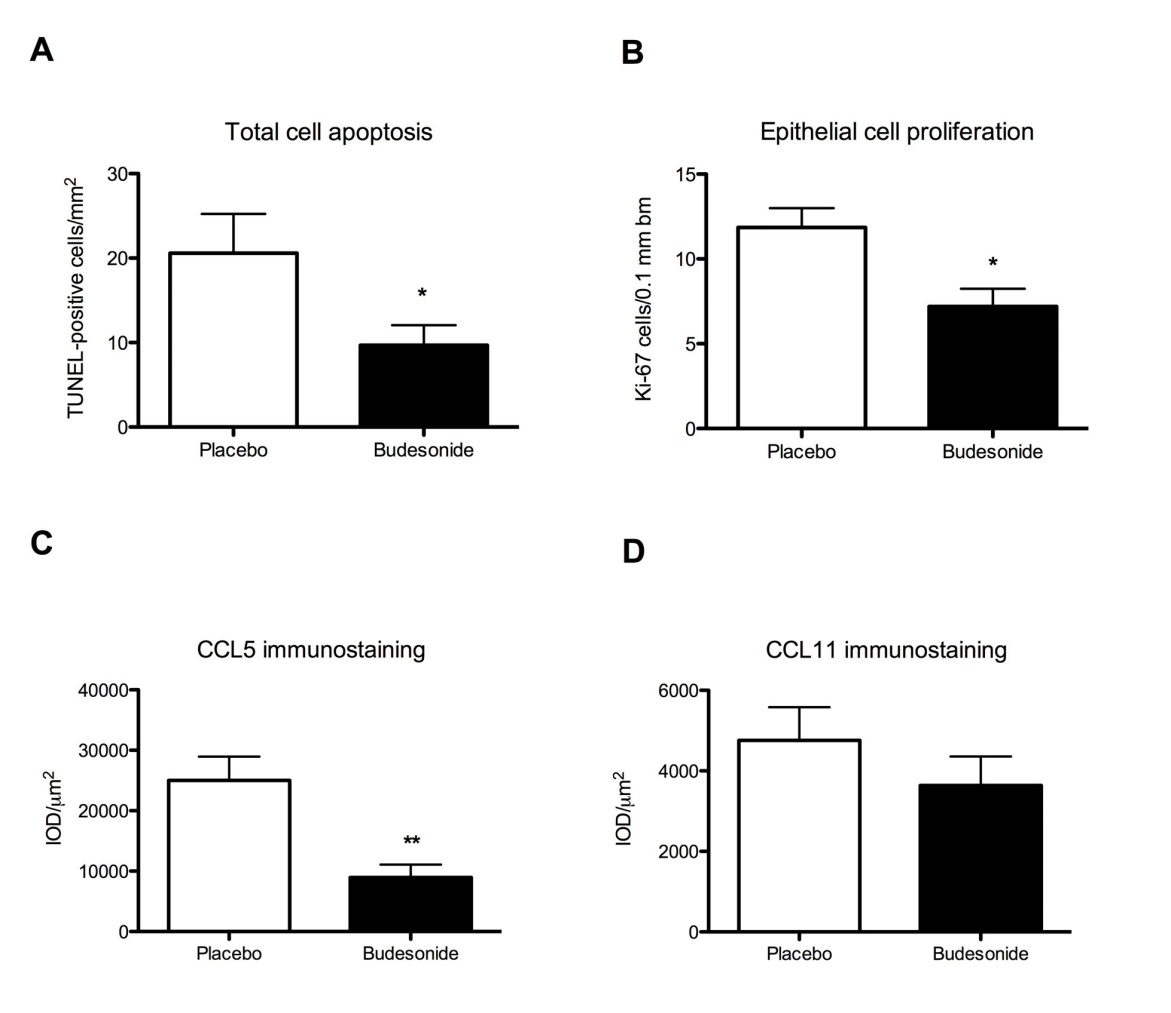
**Apoptotic and proliferating cells, and CCL5 and CCL11 staining in whole nasal biopsies at resolution of allergic inflammation**. Occurrence of apoptotic cells in the tissue quantified by TUNEL-technique (A) and occurrence of proliferating cells in the epithelium stained using the proliferating marker Ki67 and quantified in the epithelium and expressed as cells/0.1 mm basement membrane length (B) of nasal biopsies. Average integrated Optical Density (IOD) times the calculated stained area (μm^2^) reflects the occurrence of CCL5 (C) and CCL11 (D) immunostaining in nasal biopsies. Data presented is mean ± SEM. (* Denotes p < 0.05.)

The persisting allergic inflammation at placebo treatment was associated with scattered occurrence of apoptotic cells in the tissue and proliferating cells in the epithelium (Fig. 3A and 3B). These indices of inflammatory stimulus-induced local cell turnover were less pronounced in the budesonide-treated group (Fig. 3A and 3B). Furthermore, apoptotic eosinophils were detected neither in the placebo-group nor in the steroid-treated group.

### Immunostaining of CCL5 and CCL11

**Figure 4:**
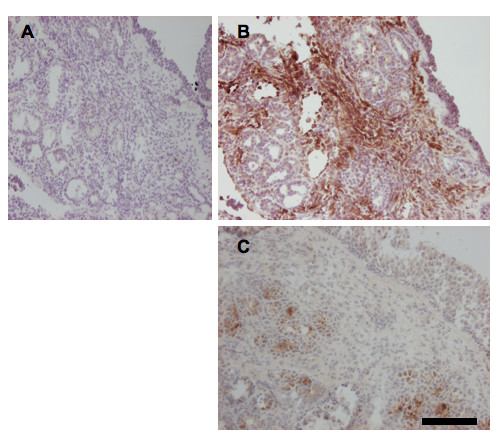
**Staining for CCL5 in whole nasal biopsies during resolution of established inflammation **Representative photomicrographs showing CCL5 immunoreactivity in the cytoplasm of cells (brown stain) counterstained with heamatoxylin (blue stain) in negative control placebo-treated individual (A) and placebo-treated (B) and steroid treated individual showing less brown staining compared to placebo treatment (C). Black scale equals 50 μm.

In placebo-treated individuals, the intensity of CCL5 immunoreactivity was pronounced in the epithelium, including the glandular epithelium, and in subepithelial tissue (Fig. 3C and 4). In steroid-treated subjects, CCL5 immunoreactivity was less than in the placebo group (Fig. 3C and 4). By contrast, the CCL11 immunoreactivity was of the same magnitude in placebo- and budesonide-treated individuals (Fig. 3D).

## Discussion

The present findings involving human airways indicate that established allergic eosinophilic inflammation are reduced by therapeutic steroid intervention already five days after institution of treatment. We further demonstrate a degree of selectivity in the steroid action in that tissue CCL5 expression, but not CCL11, is reduced by budesonide along with its attenuating effects on tissue eosinophilia. In view of the established dogma [2-4] it was most surprising that apoptotic eosinophils are not detected in the airway tissues at ongoing, spontaneous or drug-induced, resolution of the allergic airway inflammation. However, the present data obtained in a validated model of human allergic rhinitis agree with previous observations *in vivo *on early resolution of eosinophilic inflammation in steroid-treated allergic animals.

Except for observations in animal models [8] there was little prior information regarding early resolution of allergic eosinophilic airways disease *in vivo*. This study thus produced new data on the action of airway corticosteroids on eosinophilic inflammation in human airway tissues *in vivo*. Exit of inflammatory cells and proteins into the airway lumen may be a mode of elimination of these players from the inflamed tissue [9]. During resolution, increased inflammatory indices on the mucosal surface may thus be associated with corresponding reductions of the same indices in the diseased airway tissue. This complicates the interpretation of lavage fluid data in this study. Hence, the present focus was on airway tissue indices. Since the present model has a demonstrated consistency at repeated studies of symptom development and treatment effects of allergic rhinitis [19,20], we resorted to one biopsy occasion only and parallel group analyses. This was also done to avoid too much discomfort by the biopsy procedures and prevent dropouts. An additional consideration was that the institution of treatment had to be optimal to pick up early phase resolution effects and at the same time allow for a slow onset of action of the steroid treatment. This is a field where helpful precedents are scarce. As it turned out in this study, the tissue indices of eosinophilic inflammation clearly remained present in the placebo-treated subjects whereas biopsies obtained from the steroid-treated group exhibited signs of a resolution that was speeded up compared to the placebo group of patients. At allergen exposure, individuals with allergic asthma or allergic rhinitis may loose epithelial cells and the ensuing epithelial restitution processes involve increased cell proliferation [24]. It can further be expected that ongoing inflammatory processes will increase a general occurrence of cell apoptosis in the affected airway tissue. The present finding of a reduced number of apoptotic cells in airway tissues receiving therapeutic steroids thus tallies with an overall anti-inflammatory efficacy of institution of the steroid treatment [7].

It has been widely assumed that apoptosis of airway tissue eosinophils would be increased by corticosteroid treatment [2-4]. Inducement of eosinophil apoptosis (followed by a postulated efficient engulfment of the apoptotic eosinophils) has thus been advocated as a major pharmacological mechanism to bring about resolution of airway eosinophilic inflammation [2-4]. By contrast, animal *in vivo *data have scarcely supported a role of apoptosis in the pharmacology of eosinophil elimination [6,8]. Thus, similar to the present findings, apoptotic eosinophils have not been unequivocally detected in steroid-treated allergic airway tissues *in vivo*. Such negative data are strengthened by the concomitant demonstration, as in this study, that other types of apoptotic cells are observed in the allergic tissues. The present observations are remarkable because we examined a phase where the likelihood of eosinophil apoptosis should be the greatest both in spontaneous and drug-induced resolution. Our data may explain the scarcity of publicised reports on eosinophil apoptosis in airway biopsies including the lack of confirmation of a reported insignificant trend at steroid-induced increased eosinophil apoptosis in asthma [4]. Furthermore, the present observations question the support that the current dogma is getting by reference to reports on occurrence of apoptotic eosinophils in the lumen of diseased airways [4]. Indeed, airway lumen findings in this case have little relevance because apoptotic cells in the airway tissue cannot migrate and can, therefore, not be revealed in airway lumen samples. It is at present unclear if apoptosis of tissue eosinophils is a desirable drug action since poor engulfment of these cells may aggravate airway allergic inflammation [25]. A poor engulfment *in vivo *would further mean that apoptotic eosinophils, when they occur in the tissue, should be readily detected. Hence, we suggest that the present negative observations reflect true rarity of eosinophil apoptosis in spontaneously resolving as well as in corticosteroid-treated human nasal allergic airway tissues.

The present negative data on occurrence of apoptotic eosinophils in the airway tissue at resolution of inflammation support the possibility that airway tissue eosinophils can be swiftly and non-injuriously eliminated through egression into the airway lumen [9]. In the lower airways, egressed cells would be mixed with plasma exudates and airway secretions and removed by the mucociliary escalator. Removal of inflammatory cells on the airway surface would be even more functional in the nasal passages since final elimination, through swallowing or blowing of the nose, is prompt and relatively uncomplicated. Animal data suggest that corticosteroid treatment does not prevent but may permit the egression of eosinophils into the airway lumen [8]. In the present study, it was considered impractical to collect all nasal outputs during several days and nights to try and calculate egression of eosinophils during the post challenge period. However, the finding that subepithelial eosinophilia tended to be reduced more than the epithelial eosinophilia is compatible with the steroid treatment permitting traffic of eosinophils towards the lumen in airways with resolving eosinophilia.

The reduced eosinophilia in the corticosteroid group would in part reflect reduced recruitment of these cells during the period of steroid treatment. Although many locally present eosinophil chemoattractants might contribute, CCL5 and CCL11 have been pointed out as two major chemokines involved in recruiting circulating eosinophils to allergic airway tissues [26]. The present demonstration of a reduced expression of CCL5, occurring simultaneously to the reduced tissue eosinophilia, suggests that CCL5, with its proposed roles in eosinophil and lymphocyte recruitment, can be of special importance as pharmacological target. It is also possible that disappearance of CCL5 from the tissue could have contributed to migration of eosinophils into the airway lumen [27]. Currently entertained molecular actions of the anti-inflammatory steroids suggest that these drugs exert non-selective inhibition of the generation of inflammatory chemokines [26]. Hence, the mechanism behind the particular *in vivo *anti-CCL5 action of corticosteroids in mouse [8] and human (this study) allergic airways remains mechanistically challenging. Other selective actions of steroids have been demonstrated previously including observations that steroid treatment, despite its potent anti-inflammatory effects, may spare leukocyte and microvascular innate responses mobilised in relation to epithelial repair [28] and microbial defence [29]. The present inhibition of CCL5 and the association between increase in airway CCL5 and deterioration of asthma at stopping steroid treatment [17] support the view that this protein may have a central role in maintaining inflammatory processes in rhinitis and asthma.

We conclude that institution of nasal steroid treatment in subjects with established symptoms of allergic rhinitis reduces tissue indices of allergic eosinophilic inflammation within five days. Our data support a role of inhibition of CCL5-dependent cell recruitment, but challenge the dogma that eosinophil apoptosis is involved in corticosteroid-induced resolution of allergic airway inflammation.

## Competing interests

The authors declare that they have no competing interests.

## Authors' contributions

LU carried out the biopsy processing, histological staining and analysis, interpretation of data, and drafted the manuscript. CAE performed the clinical studies, acquisition of clinical data, performed the statistical analysis, and drafted the manuscript. LG supervised the clinical studies. MA, JE and LG participated in the design of the study and drafted the manuscript. CGP conceived of the study, participated in its design, and helped to draft the final manuscript. All authors read and approved the final manuscript.
